# A magnetically enabled simulation of microgravity represses the auxin response during early seed germination on a microfluidic platform

**DOI:** 10.1038/s41378-021-00331-5

**Published:** 2022-01-14

**Authors:** Jing Du, Lin Zeng, Zitong Yu, Sihui Chen, Xi Chen, Yi Zhang, Hui Yang

**Affiliations:** 1 Laboratory of Biomedical Microsystems and Nano Devices, Center for Bionic Sensing and Intelligence, Institute of Biomedical and Health Engineering, Shenzhen Institute of Advanced Technology, Chinese Academy of Sciences, Shenzhen, 518055 China; 2 Center for Medical AI, Institute of Biomedical and Health Engineering, Shenzhen Institute of Advanced Technology, Chinese Academy of Sciences, Shenzhen, 518055 China

**Keywords:** Engineering, Chemistry

## Abstract

For plants on Earth, the phytohormone auxin is essential for gravitropism-regulated seedling establishment and plant growth. However, little is known about auxin responses under microgravity conditions due to the lack of a tool that can provide an alteration of gravity. In this paper, a microfluidic negative magnetophoretic platform is developed to levitate Arabidopsis seeds in an equilibrium plane where the applied magnetic force compensates for gravitational acceleration. With the benefit of the microfluidic platform to simulate a microgravity environment on-chip, it is found that the auxin response is significantly repressed in levitated seeds. Simulated microgravity statistically interrupts auxin responses in embryos, even after chemical-mediated auxin alterations, illustrating that auxin is a critical factor that mediates the plant response to gravity alteration. Furthermore, pretreatment with an auxin transportation inhibitor (N-1-naphthylphthalamic acid) enables a decrease in the auxin response, which is no longer affected by simulated microgravity, demonstrating that polar auxin transportation plays a vital role in gravity-regulated auxin responses. The presented microfluidic platform provides simulated microgravity conditions in an easy-to-implement manner, helping to study and elucidate how plants correspond to diverse gravity conditions; in the future, this may be developed into a versatile tool for biological study on a variety of samples.

## Introduction

The gravity of Earth is a unique and constant factor throughout the entire life cycle. Plants are sessile and well adjusted to this 1 g level and evolved gravitropism 500 million years ago^1^. Consequently, an alteration of gravity can profoundly influence plant physiological processes and initiate adaptive responses^2^. As a critical regulator of all aspects of plant growth and development^3^, including seed germination^4^, auxin has been identified to be responsive to gravitational stimuli through the redistribution of auxin gradients^5^. As the downstream reflection of spatiotemporal distribution, auxin responses depend on not only metabolism but also directional cell-to-cell transportation^6^. It is important to study auxin responses under microgravity conditions, which is essential for illustrating how plants perceive environmental gravity alterations and developing plant space biology.

Understanding gravitational effects on plants requires changing the magnitude of this force. In general, the way to encounter microgravity environments is either in space or during free fall^7^. The International Space Station (ISS) and Space Shuttle provide a short period of lasting microgravity conditions during spatial missions, which are rare and costly^8^. Free fall towers can eliminate the effects of constant gravity; however, they lack the supply of continuous and long-term microgravity conditions^9^. More importantly, neither method is applicable for routine laboratory research. Therefore, instruments to simulate microgravity devices have been developed and applied to meet an increased demand for laboratory studies^10^. A random positioning machine (RPM) is a typical simulator that can mimic microgravity through a rotary system, such as a one-dimensional (1D) clinostat, two-dimensional (2D) clinostat, and three-dimensional (3D) clinostat^11^. Although an RPM can generate comparable effects of microgravity when the direction change is fast enough, the limitation is to monitor several individual samples simultaneously and in real time^12^.

The magnetic levitation technique has been used to simulate microgravity via either positive or negative magnetophoresis^13,14^. However, positive magnetophoresis levitates the subject of interest by using magnetic nanoparticles that are loaded inside of the subject or combined to its surface through ligand–receptor assays. Clearly, such pretreatment procedures prior to manipulation usually take a long time and largely restrict research and applications^15^. Conversely, negative magnetophoresis-mediated levitation can exactly simulate microgravity by counteracting gravitational force in a manner of free labeling^16^. In this technique, objects are diluted in a magnetic solution located in a magnetic field. Due to magnetic force, the paramagnetic materials suspended in the solution can generate a concentration difference over the magnetic field area; the higher the magnetic field gradient is, the higher the concentration of the paramagnetic materials. Therefore, nonmagnetic objects are triggered to move toward regions of the low magnetic field along magnetic field gradients; this phenomenon is referred to as “negative magnetophoresis”, ultimately resulting in stable magnetic levitation and the simulation of microgravity conditions^17^. To reduce the magnitude of magnetic fields, simulated microgravity can also be generated by increasing the magnetic susceptibility of ferrofluids or paramagnetic media^18^. Ferrofluids are colloidal suspensions of magnetic monodomain nanoparticles, typically magnetite (Fe_3_O_4_) or maghemite (Fe_2_O_3_), with dimensions of approximately 10 nm^19^. Previous studies have shown that plant metabolism could be influenced by ferrofluids, and that cell division and chromosome aberrations might also be affected when exposed to certain concentrations of ferrofluids^20^, suggesting that ferrofluids are not suitable for plant cultivation under simulated microgravity conditions. Gadolinium has been the most widely used paramagnetic material in magnetic resonance imaging^21^. Even though this material can cause a delayed onset toxic effect, gadolinium can also be given safely in the form of chelated Gd^3+^ ions, which still retain much of their paramagnetic ability. Recently, gadolinium-based solutions have been gradually utilized in various label-free levitation applications due to their good biocompatibility, high efficiency, and relatively low cost^22–24^.

Microfluidic platforms typically offer miniaturization, integration, automation, and parallelization of (bio)chemical processes, providing advantages of handling small volumes of liquids and well-controlled microenvironments to investigate cellular and multicellular organisms^25^. The application of microfluidics for plant science studies can have a great impact on this area with considerable social and economic impacts^26^. Novel devices such as RootChip and PlantChip have been developed for plant analysis with high spatial and/or temporal resolution in the microenvironment of plant organs, demonstrating the capability of microfluidics to overcome the constraints of conventional methods^27–30^. Microfluidic devices with the negative magnetophoresis technique have been utilized to sort nanoparticles^31^ and purify biological samples such as circulating tumor cells^32^, demonstrating the great potential of using a negative magnetophoretic microfluidic platform for plant analysis.

In this study, we establish a magnetic levitation microsystem by utilizing negative magnetophoresis in a microfluidic device in which plant seeds are cultured in situ and exposed to Gd^3+^-based microgravity conditions. We perform a set of in vivo experiments to detect plant auxin responses. The effects of various concentrations of Gd^3+^ on seed levitation are assessed, and the auxin responses to Gd^3+^-triggered microgravity are investigated simultaneously. We further explore whether auxin transportation had an effect on simulated microgravity-mediated auxin responses.

## Results

### Microfluidic device to simulate microgravity condition

**Fig. 1:**
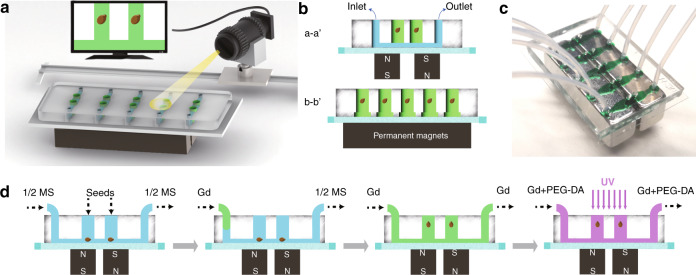
Schematic illustration of the experimental setup and procedures. **a** On-site monitoring of Arabidopsis seeds by implementing a camera on a slide toward the microfluidic device. **b** Schematic of the microfluidic chip with five channels for seed cultivation. Each channel (10 mm in length, 2 mm in width, and 100 μm in height) has two cultivation reservoirs (1000 μm in diameter, 2 mm in depth) that are cut through the PDMS layer. **c** Photograph of the microfluidic negative magnetophoresis platform. Two permanent magnets are assembled beneath the glass substrate, and the inner edges are aligned to the cultivation reservoirs. **d** The experimental procedures. First, the seeds are put into the cultivation reservoirs, and the culture medium is injected into microchannels. Second, the culture medium was replaced by Gd^3+^ solution. Third, the seeds are lifted and maintained at the position by negative magnetophoresis. Finally, PEG-DA and Gd^3+^ solutions are injected into the channels and cured by ultraviolet radiation

The on-site monitoring of Arabidopsis seeds is applied by implementing a camera on a slide toward the microfluidic device during the whole on-chip treatment procedure, including the simulated microgravity phase (see Fig. 1a). The microfluidic device is fabricated by soft lithography (details presented in the Materials and Methods section) consisting of five straight microchannels that are arranged in parallel in a polydimethylsiloxane (PDMS) layer on a glass substrate. Each microchannel has two cultivation reservoirs to independently perform the levitation process of a single seed in a reservoir. Thus, simultaneous operation of 10 seeds is enabled in a single microfluidic device. Two NdFeB permanent magnets (N52, 1″ × 1/4″ × 1/4″) with an interspace of 1000 μm are placed in different magnetic field directions beneath the glass substrate of 200 μm in thickness (see Fig. 1b, c). Due to the magnetic field gradients provided by the magnets, the paramagnetic materials suspended in the solution can generate a concentration difference over the magnetic field area. When a seed is located in the solution under magnetic field gradients, pressure is generated across its surface, levitating the seed in the solution toward the low gradient magnetic field region. The processes of seed culturing and Gd^3+^-mediated levitation are illustrated in Fig. 1d. First, Arabidopsis seeds are put into cultivation reservoirs, and culture medium (half-strength Murashige and Skoog, 1/2 MS) is injected into the microchannels by using a neMESYS syringe pump (Cetoni, Germany). The seeds are located at the bottom of the holes and cultured for 24 h. On the 2nd day, 150 mM Gd^3+^ in culture medium is introduced into the cultivation holes through the microchannels to replace the 1/2 MS medium. Once the microchannel is filled with Gd^3+^ solution, seeds are observed to gradually levitate to an equilibrium position in each cultivation reservoir. After maintaining the seeds at the equilibrium position for 30 min, the Gd^3+^ solution is then replaced by a mixed solution of Gd^3+^ and 6% (v/v) poly(ethylene glycol)-diacrylate (PEG-DA) 575, and the chip is irradiated with ultraviolet rays to solidify the mixed solution; thus, the levitated seeds are immobilized in the solution for the subsequent slicing process.

### Working mechanism of magnetic arabidopsis seed levitation

**Fig. 2:**
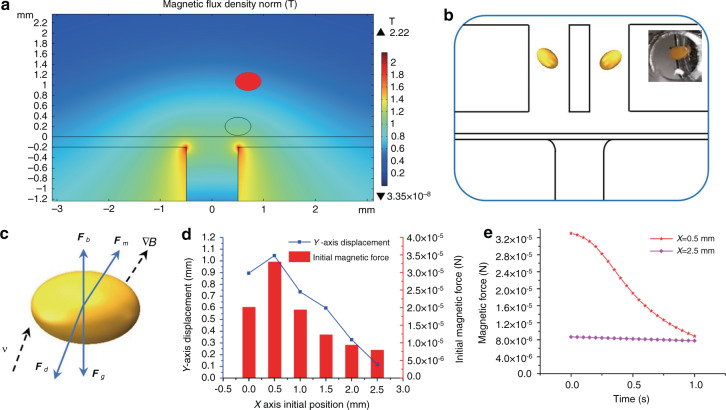
System optimization for seed levitation. **a** Simulation of seed levitation using COMSOL Multiphysics. **b** The cultivation reservoirs are designed and fabricated at the optimal positions for magnetic levitation in the chip. **c** The force analysis of the seed in Gd^3+^ solution. **d** The simulation results of initial magnetic forces and y-axis displacements of seeds starting from different *x*-axis positions. **e** The comparison of the changes in magnetic force overtime on the seed that is located at the optimal position or other positions

To cultivate the seeds in the best suspension state, a finite element method (FEM) study is performed to investigate the magnetic field distribution in the device. A numerical study on the magnetic force and moving trajectory of a seed in the magnetic solution is carried out by FEM using COMSOL Multiphysics software (COMSOL 5.4, COMSOL). The position of the cultivation reservoirs is optimized based on the simulation results (see Fig. 2a, b). In the model, the seed is simplified to an ellipsoidal shape (*a* = 250 μm, *b* = 175 μm, *c* = 175 μm, where *a* represents the major axis semidiameter; *b* and *c* represent minor axis semidiameters), the initial vertical position of its center is set as 200 μm (*Y* = 200 μm), and the initial horizontal position is changed from 0–2500 μm (*X* = 0–2500 μm). The density of Arabidopsis seeds is measured (see Supplementary Table S1 in the Supplemental Information). The seeds in the Gd^3+^ solution are subjected to the magnetic force ${{F}_{m}}$, hydrodynamic drag force *F*_d_, gravity *F*_g_ and buoyancy *F*_b_ (see Fig. 2c). The magnetic force is the main power to levitate the seed, which is given by

1 $$F_m = \frac{{V\Delta \chi }}{{\mu _0}}\left( {B \cdot \nabla } \right)B$$

where *B* is the magnetic induction intensity at the location of the seed, *μ*_0_ is the permeability of free space, *V* is the volume of the seed, and ${\Delta}{\chi}$ is the difference in magnetic susceptibility between the seed and the Gd^3+^ solution. The main factors that determine the magnitude of the magnetic force are the magnetic field intensity and the magnetic field gradient. The simulation results show that near the edges of the two magnets, both the magnetic field intensity and the magnetic field gradient reach the maximum, so the magnetic force along the magnet edge (*X* = ± 500 μm) is greater than that at other positions, and in this case, the seed can be levitated by the magnetic force to the largest displacement (see Fig. 2d). However, the magnetic force and the levitation height decrease tremendously when the seed moves away from the magnet edge, as shown in Fig. 2e. Therefore, cultivation reservoirs are designed and fabricated in a microfluidic channel at positions above the two magnet edges.

### Auxin response to the simulated microgravity condition

**Fig. 3:**
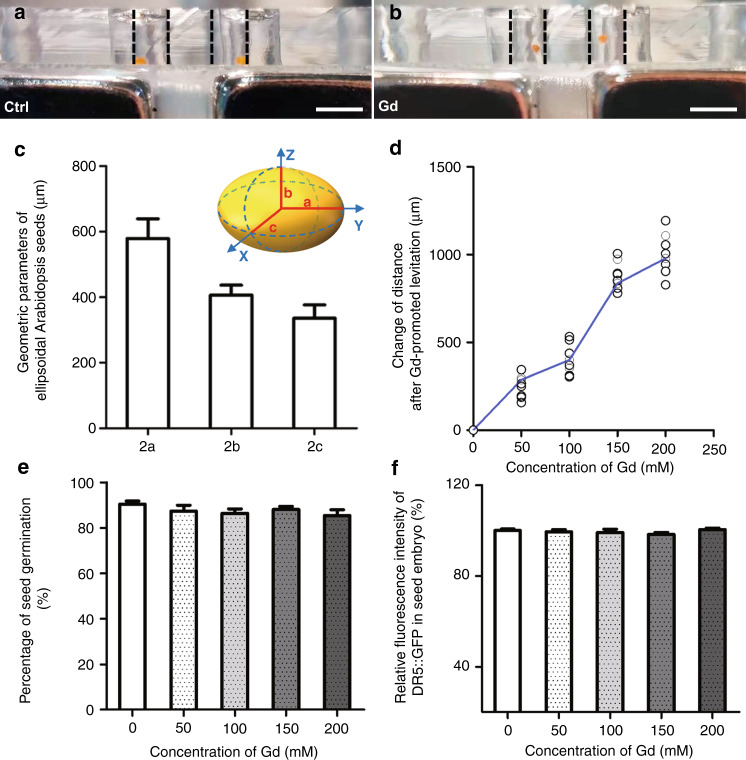
Assessment of germination and auxin distribution of Arabidopsis seeds by optimization of Gd^3+^-promoted magnetic levitation in the device. **a** Location of the seeds in the cultivation reservoirs with 1/2 MS medium (Ctrl). **b** The levitated seeds in the Gd^3+^ solution with magnets. Black dotted lines indicate the edges of reservoirs. Scale bar = 1000 μm. **c** Quantification of geometric parameters of Arabidopsis seeds. Data are the mean values ± standard error (SE), with three biological repeats for each sample (*n* = 30). **d** Histograms of the change in the levitation height for the seeds in various concentrations of Gd^3+^ solutions. Each dot represents one seed in the device. **e** Quantification of the percentage of seed germination after treatment in the indicated Gd^3+^ solutions for 30 min. Data are the mean values ± SE, with three biological repeats for each sample (*n* = 45). **f** Quantification of the relative fluorescence intensity of DR5::GFP in Arabidopsis embryo sections. Data are the mean values ± SE, with three biological repeats for each sample (*n* = 30)

Arabidopsis seeds are located at the bottom of the reservoirs on the microfluidic device when the culture medium is introduced (see Fig. 3a) and eventually float when the Gd^3+^ solution is added to the microfluidic channels (see Fig. 3b). To detail the ellipsoid-like seeds, the semidiameters *a*, *b*, and *c* are measured and calculated according to microscopic images. After statistical analysis, the lengths along the x, y and z axes, i.e., 2*a*, 2*b*, and 2*c*, are 578.0 ± 60.6 μm, 406.1 ± 30.5 μm, 335.6 ± 40.7 μm, respectively (see Fig. 3c). Then, seed quality is evaluated by the germination rate (details presented in Supplemental Information Section 1). To guarantee complete levitation, the seed needs to be lifted by a distance at least over the semidiameter of the major axis, i.e., >300 μm. The optimal concentration of the Gd^3+^ solution is 150 mM to ensure this levitation distance, and the latter can be slightly improved when the concentration of the solution increases to 200 mM (see Fig. 3d). Thus, 150 mM is sufficient to create an accelerated microgravity niche for Arabidopsis seeds on the chip. More importantly, the toxicity of Gd^3+^ solution on seed germination is tested. The experimental results show that the seeds immersed in Gd^3+^ solution for 30 min present limited differences from untreated seeds during the subsequent germination process (see Fig. 3e). Therefore, the Gd^3+^ solution with a concentration of 150 mM is safe and efficient for the study of seed response and development under simulated microgravity conditions. Moreover, it should be noted that Arabidopsis seeds are nonmagnetic. The magnetic field only acts on the magnetic Gd^3+^ solution to generate a concentration difference on the magnetic molecules so that the seed levitation is due to collisions caused by the thermal motion of magnetic molecules. Therefore, the magnetic field generated in this magnetic levitation system does not affect further biological studies on Arabidopsis seeds.

**Fig. 4:**
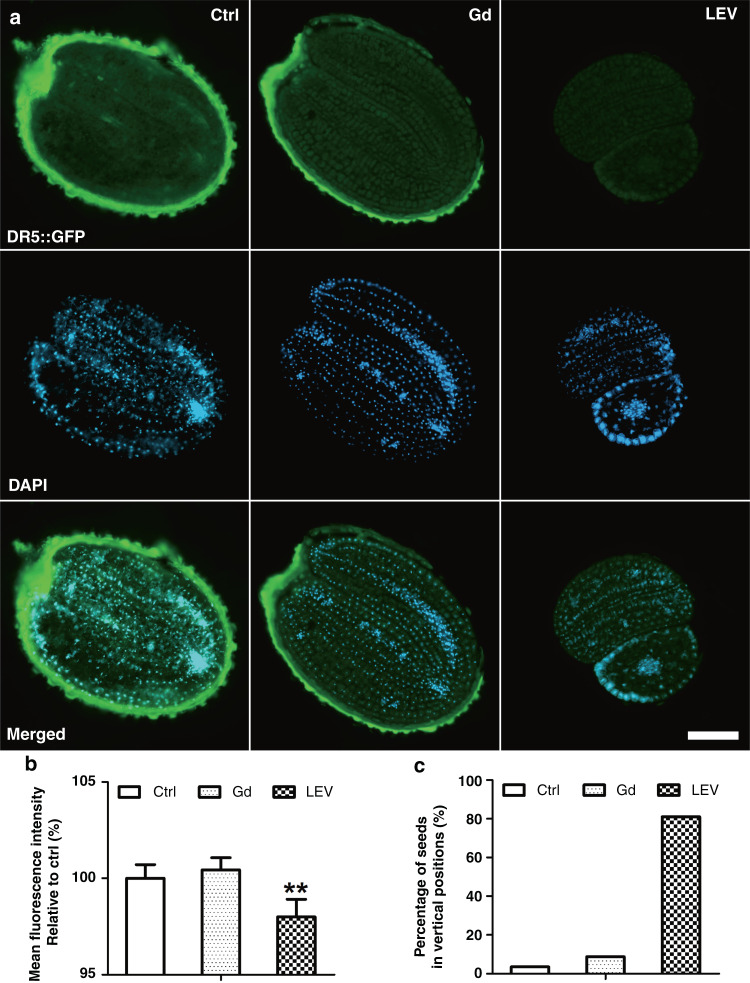
Magnetic levitation represses the auxin response during Arabidopsis seed germination. **a** Expression of DR5::GFP in embryo sections of Arabidopsis seeds without (Ctrl) and with magnetic levitation (LEV)-stimulated microgravity. Blue indicates fluorescence from 4´,6-diamidino-2-phenylindole (DAPI) dye, green indicates GFP, and the oval outline in strong green indicates autofluorescence from seed coats. Scale bar = 100 μm. **b** Quantification of the relative expression of DR5::GFP in embryo sections of Arabidopsis seeds. Error bars represent SE of the mean with three biological repeats (*n* > 30). Asterisks indicate significant differences (Student’s *t* test, **P ≤ 0.01). **c** Quantification of the percentage of Arabidopsis seeds in 2D vertical positions. Over 50 seeds are used and analyzed for each treatment as defined in (**a**)

Auxin is an essential phytohormone that contributes to all aspects of plant development^33^. It is well established that auxin-triggered root morphology and global gene expression patterns change considerably when Arabidopsis plants are grown in a spaceflight environment^34^. However, gene expression from whole plant roots or seedlings^35–37^ has difficulty on indicating diversity at the individual cell level^38^. Here, we study the auxin response during early seed germination under stimulated microgravity conditions by negative magnetophoresis. To immobilize the levitated seeds in a timely manner, poly(ethylene glycol)-diacrylate (PEG-DA)-mediated polymerization is performed, and the procedure is optimized (details presented in Supplemental Information Section 2). The levitated Arabidopsis seeds are immobilized in the polymerized hydrogel, which are then cryostat sliced for cellular images. Here, transgenic plant DR5::GFP is selected as an indicator of the auxin response, as illustrated by the fluorescent signal of DR5 promoter-driven GFP proteins. In the experiments, the influences on the auxin signal during early germination by short-term treatment with Gd^3+^ could be neglected, as shown in Fig. 3f. Taking Arabidopsis seeds in 1/2 MS medium as a control, the auxin response of DR5::GFP in Gd^3+^ solution represents similar fluorescence intensity, suggesting that Gd^3+^ hardly affects the auxin response and distribution within half an hour (see Fig. 4a). Furthermore, by replacing 1/2 MS medium with the Gd^3+^ solution on the microfluidic device, the seeds are levitated and treated under-stimulated microgravity conditions for 30 min. The auxin response is then significantly repressed (see Fig. 4b). Moreover, the levitated seeds appear to be lifted to different equilibrium positions in various orientations. Approximately 80% of these seeds are vertically levitated, compared to most seeds maintaining horizontal positions in the control condition (see Fig. 4c). The numerical simulation also illustrates vertical and horizontal displacements of the seed due to the resultant transient force, indicating seed rotation during the levitation process (see Fig. 2b). Both experimental and simulation results reveal the moving trajectory of the seed before it reaches the equilibrium position, and the auxin response is dramatically reduced under magnetic levitation conditions.

The auxin response is the downstream reflection of the spatiotemporal distribution of auxin, depending on not only its metabolism but also its directional cell-to-cell transportation^39^. To further study the role of auxin dosage in the regulation of reduced auxin responses after magnetic levitation, the auxin donor indole-3-acetic acid (IAA) and competitive inhibitor TAA1-dependent auxin synthesis inhibitor L-kynurenine (L-Kyn) are utilized. The treatment conditions of these chemicals are optimized, and details are presented in Supplemental Information Section 3.

**Fig. 5:**
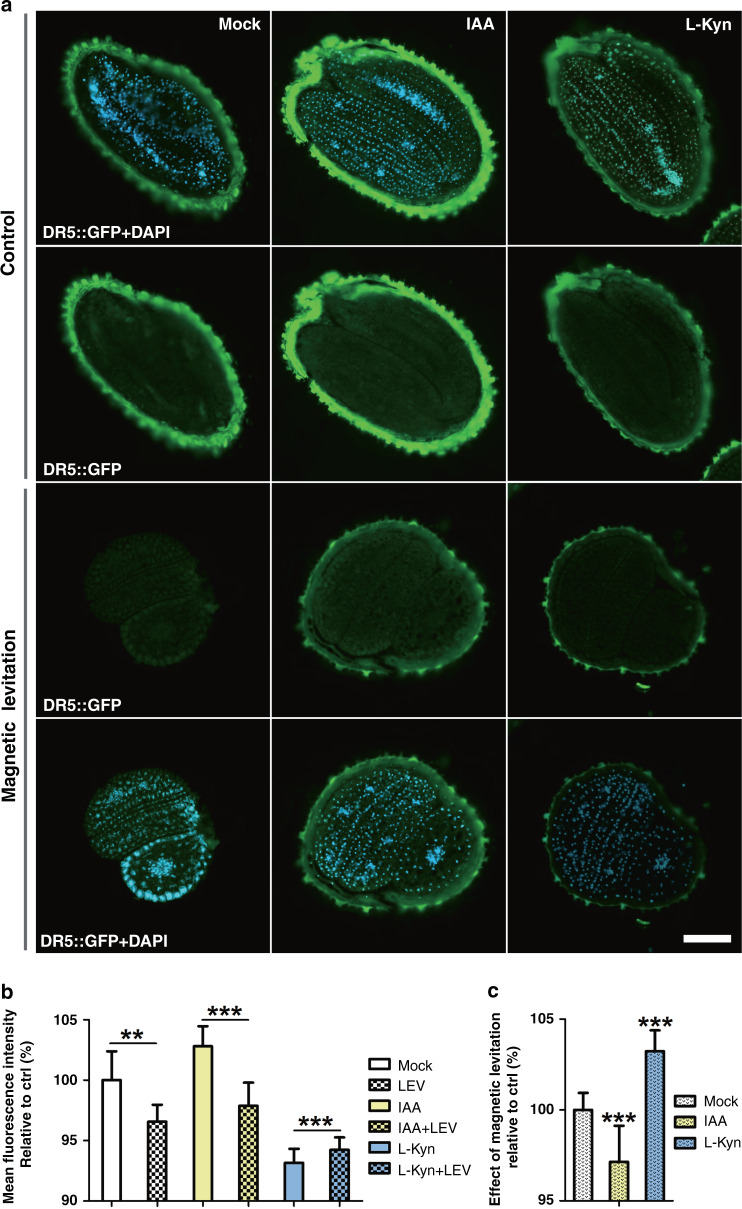
The magnetic levitation-mediated auxin response is regulated by auxin distribution in Arabidopsis embryos. **a** Embryo sections of DR5::GFP seeds treated with mock (Mock), 500 μg/L L-Kyn for 12 h or 100 μg/L IAA for 30 min in advance (Control), while expression of DR5::GFP in seed embryos treated with Gd^3+^-induced magnetic levitation (LEV) for 30 min. Scale bar = 100 μm. **b** Quantification of the relative expression of DR5::GFP in Arabidopsis embryos after IAA or L-Kyn treatment without (blank rectangle) and with Gd^3+^-induced LEV (filled rectangle with dots). **c** Quantification of the percentage of DR5::GFP cells with Gd^3+^-induced LEV divided by those without mock, IAA or L-Kyn treatment as defined in (**a**)

To evaluate the impact of Gd^3+^ on auxin-related chemicals, the auxin response is examined first with the addition of the chemicals and Gd^3+^. After pretreatment of seeds with either IAA or Kyn, the cellular fluorescent signal is increased or reduced accordingly (see Fig. 5a). Under magnetic levitation conditions, the auxin responses to these two chemicals are significantly decreased. When the seeds are pretreated with IAA, the auxin response inside the seeds is highly reduced during magnetic levitation (see Fig. 5b). L-Kyn effectively suppresses the auxin response in Arabidopsis seeds, and the levitation condition cannot inhibit the auxin response further, but a slight increase is observed (see Fig. 5c), indicating that the levitation condition shows a limited effect on the auxin response if the auxin level is low. It was reported that L-Kyn is a specific and highly effective inhibitor of the TAA1/TAR pathway in TAA1/TAR-mediated auxin biosynthesis, and L-Kyn can target only the TAA1/TAR family of plant amino-transferases but not other related families^40^. In our experiment, with pretreatment of L-Kyn, LEV does not repress auxin levels, suggesting that LEV-mediated auxin alteration occurs through the L-Kyn-involved TAA1/TAR pathway. Moreover, compared to L-Kyn pretreatment alone, LEV treatment slightly increases the auxin level. Since auxin often forms a positive feedback loop to increase its own synthesis, the cooperation of L-Kyn and LEV may trigger this positive feedback mechanism^40–42^.

Data are the mean values ± SE, with three biological repeats for each sample (*n* = 45). Asterisks indicate significant differences (Student’s *t* test, **P ≤ 0.01; ***P ≤ 0.001).

**Fig. 6:**
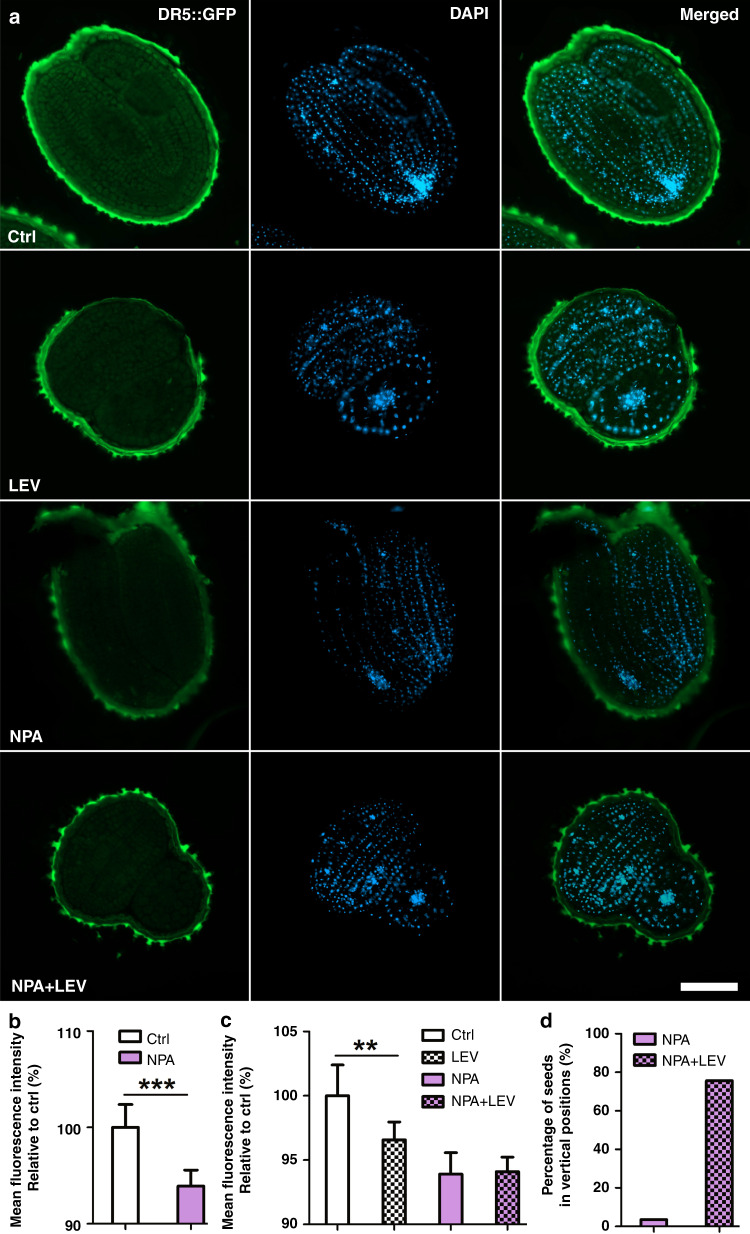
Auxin transportation has a greater impact on the auxin response in Arabidopsis embryos during magnetic Gd^3+^-induced seed levitation. **a** Expression of DR5::GFP in Arabidopsis seed embryos without (Ctrl) or with 500 μg/L NPA pretreatment for 12 h (NPA) following treatment with Gd^3+^-induced LEV for 30 min (LEV or NPA + LEV). Scale bar = 100 μm. **b** Quantification of the expression of DR5::GFP with or without 500 μg/L NPA treatment. Data are the mean values ± SE, with three biological repeats for each sample (*n* = 30, Student’s t test, ***P ≤ 0.001). **c** Quantification of the percentage of Arabidopsis seeds in vertical positions. Over 30 seeds are analyzed for NPA-pretreated samples without or with Gd^3+^-induced LEV. **d** Quantification of the expression of DR5::GFP in Arabidopsis seed embryos as defined in (**a**). Data are the mean values ± SE, with three biological repeats for each sample (*n* = 30, Student’s *t* test, **P ≤ 0.01)

To further study the contribution of auxin transportation during the levitation process, the auxin transportation inhibitor N-1-naphthylphthalamic acid (NPA) is used to pretreat the seeds. As shown in Fig. 6a, b, NPA significantly suppresses the auxin response in seeds, and the fluorescent signal of DR5::GFP is not further reduced under magnetic levitation conditions compared to the results obtained under untreated conditions (see Fig. 6c). Therefore, the levitation-mediated alteration of the auxin response is mainly regulated by auxin transportation. Moreover, levitation-triggered repositioning is hardly affected by the NPA treatment (see Fig. 6d), as the group treated with NPA and levitation shows equivalent percentages compared to the group treated only with levitation (see Fig. 4c). Together, auxin transportation has significant effects on auxin responses during negative magnetophoresis-mediated seed levitation.

## Discussion

Auxin has been established as one of the critical factors mediating plant growth and development. However, due to the difficulties in generating microgravity conditions on Earth, the commonly used microgravity simulator in the lab cannot provide spatiotemporal observations of plants at the cellular level. Here, we design and fabricate a microfluidic device for the long-term culturing and monitoring of Arabidopsis seeds in situ. With the application of Gd^3+^-triggered negative magnetophoresis, plant seeds are levitated on the device to simulate microgravity conditions.

It is well established that auxin-triggered root morphology and global gene expression patterns change considerably when Arabidopsis plants are grown in a spaceflight environment^34^. However, gene expression from whole plant roots or seedlings^35–37^ has difficulty on indicating diversity at the individual cell level^38^. In this study, with the benefit of magnetic levitation and PEG-DA 575-mediated photopolymerization to solidify levitating seeds, the auxin response of the overall plant embryo can be distinguished at the cellular level. Seed sections are further examined with an obvious decrease. Moreover, Gd^3+^-promoted simulated microgravity repression of the auxin response is mainly mediated by auxin transportation but is unaffected by the biosynthesis pathway within half an hour.

In the future, the mechanism of the auxin response to microgravity in plants can be further studied. Moreover, due to the straightforwardness of this approach, the microfluidic negative magnetophoresis platform will be a robust and versatile tool, facilitating studies on a variety of biological objects to exploit their unique properties under microgravity conditions.

## Materials and methods

### Design and fabrication of the microfluidic negative magnetophoresis (MNM) platform

**Fig. 7:**
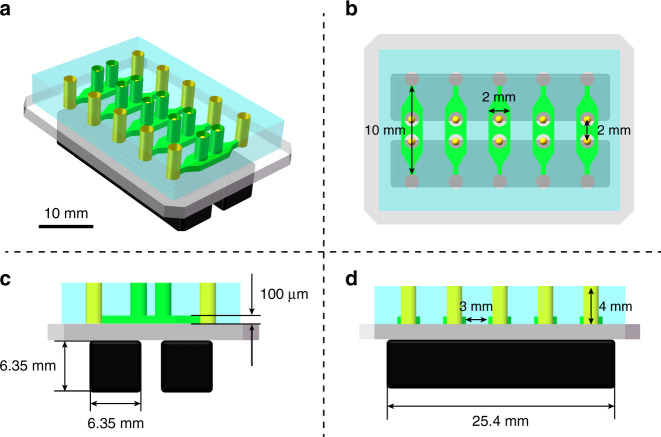
Design of the MNM platform to simulate microgravity conditions for Arabidopsis seeds. **a** Three-dimensional view of the MNM platform. Scale bar = 10 mm. **b** Top view of the chip with five microchannels for seed cultivation. **c** Layout of the microdevice showing the setting of each microchannel and the magnets. **d** Layout of the microdevice with the position of five inlets and magnets

**Fig. 8:**
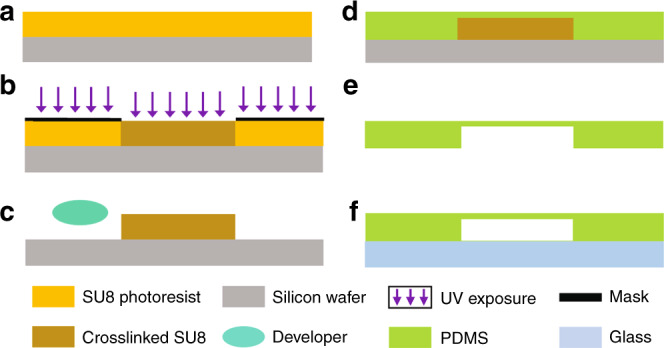
Schematic illustration of the fabrication process of the microfluidic chip. **a** The silicon wafer is spin-coated with SU-8. **b** SU-8 is patterned by UV exposure. **c** The SU-8 structure is obtained after the development process. **d** PDMS is casted on the SU-8 master. **e** The PDMS layer is peeled off from the master. **f** The PDMS layer and the glass substrate are bonded by oxygen plasma treatment

Silicon wafers and fused silica glass wafers (Ø4 in., 500 μm in thickness) were purchased from RDMICRO (China). SU-8 photoresist and its developer were purchased from MicroChem (USA). Positive photoresist (S1805 and AZ4620) and developer were purchased from Rohm and Haas Electronic Materials (USA) and from AZ Electronic Materials (China), respectively. Polydimethylsiloxane (PDMS) prepolymer and curing agent were purchased from Dow Corning (USA). The microfluidic device consisted of a PDMS layer and a glass substrate. Five parallel microchannels with a total of ten cultivation reservoirs on the PDMS layer were cast from an SU-8 structure that was fabricated on a silicon wafer by soft lithography. The MNM platform was realized by assembling the microfluidic device with two permanent magnets (see Fig. 7a). The inner edges of the two magnets were aligned to the center of the cultivation reservoirs, where both the magnetic field strength and the magnetic field gradient reached the maximum according to numerical analysis. The details of the MNM platform in a 2D view are presented in Fig. 7b, d. The whole fabrication process is shown in Fig. 8. Finally, the device was connected to a syringe pump (neMESYS, Cetoni, Germany) for further experiments.

The microfluidic device was fabricated by plasma bonding between a PDMS layer with microfluidic structures and a glass substrate. The PDMS layer was fabricated by soft lithography. In brief, a 180 μm thick layer of SU-8 2100 (MicroChem Corp.) was spin-coated onto a silicon wafer and soft baked at 95 °C for 35 min. The wafer was then exposed to the photomask containing the chip design at 280 mJ/cm^2^ using an EVG mask aligner (610TB, EV Group GmbH). After the postexposure bake at 95 °C for 13 min, the wafer was placed in a dish of SU-8 developer for approximately 12 min and gently agitated until the exposed features remained. The wafer was rinsed with clean IPA and then dried with N_2_ gas. The master was then used to mold PDMS (Sylgard 184, Dow Corning Corp.). PDMS was cast at a 1:10 ratio (curing agent/base elastomer), degassed under vacuum, and cured in an oven for 2 h at 85 °C. Access holes and cultivation reservoirs were punched on the PDMS layer, which was then completely cleaned for oxygen plasma bonding.

### Modeling and simulation

The numerical study of the negative magnetophoretic effect on Arabidopsis seeds was carried out by FEM. The seeds immersed in Gd^3+^ solution experience a negative magnetophoretic force *F*_m_, hydrodynamic drag force *F*_d_, gravity *F*_g_ and buoyancy *F*_b_, and the governing equations are

2 $$F_{\mathrm{m}} = \frac{{V_{s}\left( {\chi _{\mathrm{s}} - \chi _{\mathrm{m}}} \right)}}{{\mu_{0}}}\left( {B \cdot \nabla } \right)B$$

where *μ*_0_ is the permeability of free space, *V*_s_ is the volume of seed, and *χ*_s_ and *χ*_m_ are the magnetic susceptibilities of the seed and the Gd^3+^ solution (381667, Sigma), respectively. The relationship between magnetic susceptibility and paramagnetic salt concentration is given by Eq. (3)^43^:

3 $$\chi _{\mathrm{m}} = \left( {0.35 \times C_{\mathrm{Gd}} - 9.04} \right) \times 10^{ - 6}$$

where *C*_Gd_ is the concentration of Gd^3+^ solution (mM).

The hydrodynamic drag force *F*_d_ is given by Eq. (4):

4 $$F_{\mathrm{d}} = 6{\uppi \upeta _{\mathrm{s}}}{\mathrm{r}}\Delta {\mathrm{vf}}_{\mathrm{D}}$$

where r is the radius of the particle (here, the semidiameter of the major axis a of the ellipsoid is used instead), Δv is the velocity difference between the seed and the Gd^3+^ solution, η_s_ is the viscosity of the Gd^3+^ solution, *f*_D_ is the drag coefficient of the particle, and the value incorporates the influence of a solid wall in the vicinity of the moving seed, given by Eq. (5):

5 $$f_D = \left[ {1 - \frac{9}{{16}}\left( {\frac{r}{{r + z}}} \right) + \frac{1}{8}\left( {\frac{r}{{r + z}}} \right)^3 - \frac{{45}}{{256}}\left( {\frac{r}{{r + z}}} \right)^4 - \frac{1}{{16}}\left( {\frac{r}{{r + z}}} \right)^5} \right]^{ - 1}$$

where *z* is the distance of the seed to the solid wall.

The gravity *F*_g_ is given by Eq. (6):

6 $${\mathrm{F}}_{\mathrm{g}} = {\mathrm{m}}_{\mathrm{s}}{\mathrm{g}}$$

where m_s_ is the weight of a single seed and g is the gravitational constant.

The buoyancy *F*_b_ is given by Eq. (7):

7 $$F_{\mathrm{b}} = \rho _{\mathrm{m}}gV_s$$

where *ρ*_m_ is the density of the Gd^3+^ solution.

In the simulation, the “magnetic fields, no current” module was used to solve the magnetic field distribution around the seed, and the magnetic force was calculated based on Eq. (1) and then based on the governing equation:

8 $$m_{{{\mathrm{s}}}}\frac{{{{{\mathrm{d}}}}u_{{{\mathrm{p}}}}}}{{{{{\mathrm{d}}}}t}} = F_{{{\mathrm{m}}}} + F_{{{\mathrm{d}}}} + F_{{{\mathrm{L}}}}$$

The “laminar flow” module, the “solid mechanics” module, and the “fluid-structure interaction” module were used to solve the seed moving trajectories in Gd^3+^ solutions. where *m*_s_ and *u*_p_ are the mass and velocity of the seeds, respectively. We used the FEM to build the simulation model in COMSOL Multiphysics (COMSOL 5.4, COMSOL Inc.). The magnetic field distribution over the levitation area was thoroughly studied, and the moving trajectory of the seeds in the Gd^3+^ solution was obtained based on the above force analysis.

### Plant growth and seed harvest

The Arabidopsis wild-type and transgenic plants used in this study were in a Col-0 (Columbia) background ordered from Nottingham Arabidopsis Stock Centre (NASC). All plants were grown in a controlled growth chamber (MGC-100HP-2L, Shanghai Yiheng Technical Co., Ltd.) at 20–22 °C under cool-white fluorescent light (80–100 μmol m^−2^ s^−1^) under a long-day photoperiod (16 h light/8 h dark). Arabidopsis seeds were harvested only after the siliques had completely browned.

### Seed stratification and germination

The surface of Arabidopsis seeds was sterilized using 50% (v/v) bleach solution in tubes^44^. After sterilization, seeds were suspended in autoclaved distilled water and then kept at 4 °C in a refrigerator for 2 days to break seed dormancy. After refrigerated stratification, seeds were sown in cylindrical reservoirs (1000 μm in diameter) on-chip and cultured with half-strength Murashige and Skoog (1/2 MS) medium for 1 day.

### Cryostat slicing and fluorescent labeling

After maintaining the seeds at the equilibrium position of magnetic levitation, a mixture of Gd^3+^ and PEG-DA575 was used to replace the levitation medium and solidified by ultraviolet rays. The solidified levitating seeds were carefully removed using a blade to remove the solidified medium. The seeds were then embedded in an optimal cutting temperature compound (OCT, 23-730-571, Fisher HealthCare) in a container. Then, OCT-embedded seeds were snap-frozen and stored in the container at −80 °C in a refrigerator for at least 2 h. Sliced sections (25 μm) were cut from one side of the seeds to the opposite side continuously using a cryostat (CM1950, Leica). From approximately 20 slides per seed, each slide was placed on a glass slide and dried at room temperature. These slides were then washed with phosphate-buffered saline (PBS, 1×, pH 7.4) buffer to remove OCT and fixed with paraformaldehyde (PFA, 158127, Sigma) for 10 min. After washing in PBS buffer again, the slices were mounted with DAPI solution (10236276001, Sigma) and sealed with cover glasses. DAPI is usually utilized as an obvious indicator of the nucleus in cells. In this study, DAPI staining was used to locate the plant nucleus and indicated the location of plant cells for the distinction of plant-embryonic cells. Thereafter, the slices were imaged using a Zeiss Axio Observer 7 inverted fluorescence microscope (Carl Zeiss Microscopy GmbH) equipped with light-emitting diodes, appropriate fluorescent filter sets, and an sCMOS camera (ORCA-Flash 4.0-V3, Hamamatsu). Images were exported through Zen software (Zen 3.1, Zeiss).

## Supplementary information

Supplemental Information

Graphical abstract
