# Supplementary material for: A magnetically enabled simulation of microgravity represses the auxin response during early seed germination on a microfluidic platform
Source: Microsyst Nanoeng. 2022 Jan 14;8:11. doi: 10.1038/s41378-021-00331-5 (PMC8760315; doi:10.1038/s41378-021-00331-5)
Supplement: Supplementary file 1 — Supplemental Information [file 41378_2021_331_MOESM1_ESM.docx]

Magnetically enabled simulation of microgravity represses auxin response during early seed germination on a microfluidic platform

Jing Du ^a^, Lin Zeng ^a^, Zitong Yu ^a^, Sihui Chen ^a^, Xi Chen ^a^, Yi Zhang ^b^ and Hui Yang ^*,a^

1. Laboratory of Biomedical Microsystems and Nano Devices, Center for Bionic Sensing and Intelligence, Institute of Biomedical and Health Engineering, Shenzhen Institute of Advanced Technology, Chinese Academy of Sciences, Shenzhen 518055, CHINA.
2. Center for Medical AI, Institute of Biomedical and Health Engineering, Shenzhen Institute of Advanced Technology, Chinese Academy of Sciences, Shenzhen 518055, CHINA.

*Correspondence should be addressed to Hui Yang; [hui.yang@siat.ac.cn](mailto:email@institution.edu)

**Section 1. Evaluation of seed quality**

There are a number of quick tests for assessing seed quality and its germinability [1]. Due to difficulties of handling small size of Arabidopsis seeds, visual inspection, seed sectioning, or flotation testing is inadaptable. To evaluate Arabidopsis seed quality, seed germination is the most reliable method to determine seed viability. After seed stratification process, Arabidopsis seeds were cultured on 1/2 MS medium supplementary with 1% sucrose and 0.8% agar for 2 days. On the 2^nd^ day, most of radicals were merged from seed hole. According to statistical analysis, around 90% of transgenic plant DR5::GFP germinated, similar as that in the wild-type (Supplementary Figure 1). Equivalent high germination rate of DR5::GFP and wild-type seeds indicated good seed quality.


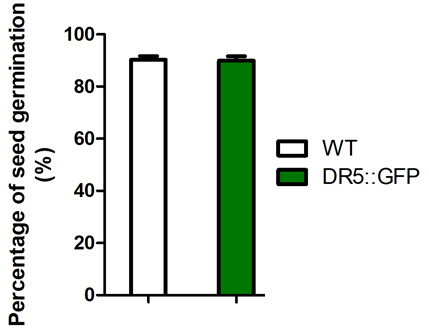


**Supplementary Figure 1.** Quantification of percentage of Arabidopsis seed germination in the wild-type (WT) and DR5::GFP of Columbia-0 ecotype (Col-0). Error bars represent standard error (SE) of the mean. (n>100, three biological repeats).

**Section 2. Immobilization of seed levitation for cryostat slicing**

To immobilize the status of negative-magnetophoresis induced seed levitation, poly(ethylene glycol)-diacrylate (PEG-DA) hydrogel based polymerization was utilized. PEG-DA200 and PEG-DA575 (average molecular weight of ~200 and ~575) with the difference in the number of oxyethylene units [2] were chosen. A series of concentrations were used and optimized for PEG-DA mediated polymerization. Without UV treatment, the liquid in 96-well plate was clear. Under the UV exposure, the liquid inside each well turned to cylinder-shaped solid. Compared to seldom solidification of PEG-DA 200 after UV exposure, PGE-DA 575 mediated photo-polymerization was much better. Visually, polymerization was made quite completely when the percentage of PEG-DA 575 reached 4% and above (Supplementary Figure 2A). To optimize this phenomenon, various concentrations（from 4% to 8%）were further tested. All the concentration of PEG 575 can form solidified hydrogel. Moreover, 4% and 5% of PEG 575 polymerized into soft shape, while 8% hydrogel represented hard and less transparent (Supplementary Figure 2B). Taken results together, 6% of PEG-DA575 is an optimized concentration for UV-mediated photo-polymerization and can be used for solidification of the levitated Arabidopsis seeds (Supplementary Figure 3). Seeds merged in polymerization hydrogel were immediately immersed in Optimal Cutting Temperature (OCT) compound and kept at - 80 ℃ [refrigerator](C:/Users/DUJING/AppData/Local/youdao/dict/Application/8.9.3.0/resultui/html/index.html#/javascript:;) to prepare following cryostat slicing. The nuclei of embryo cells on the slices were illustrated by 4′,6-diamidino-2-phenylindole (DAPI) staining. The integrated section of seeds was easily viewed by the differential interference contrast (DIC) channel on the microscope and the cellular nuclei were clearly visualized by using fluorescent dye (Supplementary Figure 4).


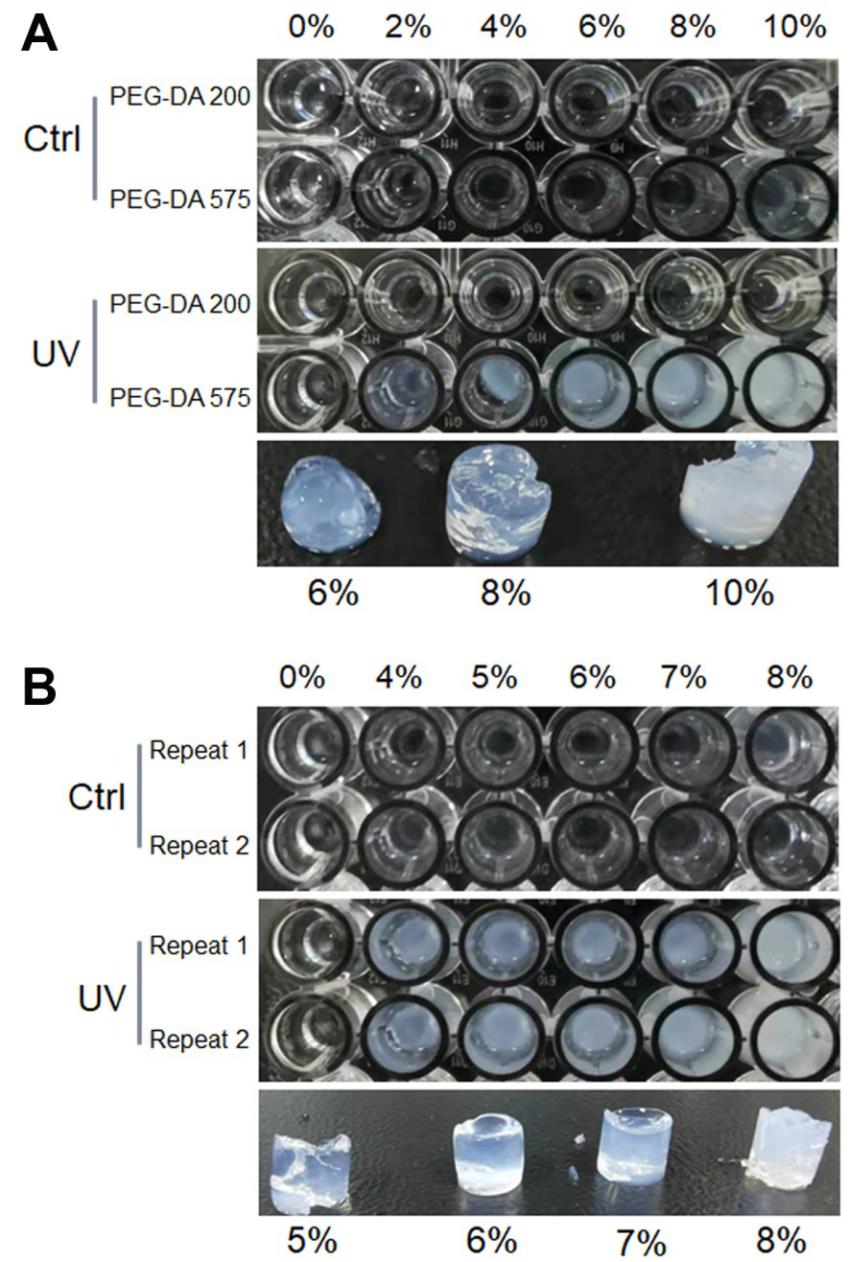


**Supplementary Figure 2.** UV-initiated photo-polymerization of PEG-DA hydrogel. (A) Photo-polymerization of PEG-DA 200 and PEG-DA 575 for the indicated concentrations without (Ctrl) and with UV exposure (UV) for 30 seconds in 96-well plate. The cylinder-shaped polymerized PEG-DA 575 at the indicated concentration was placed in the last row, respectively. (B) Photo-polymerization of PEG-DA 575 at varying concentrations (2 replicates), without (Ctrl) and with UV exposure (UV). The appearance of cylinder-shaped polymerized PEG-DA 575 (varying from 5% to 8%, v/v) was visually checked and presented in the last row.


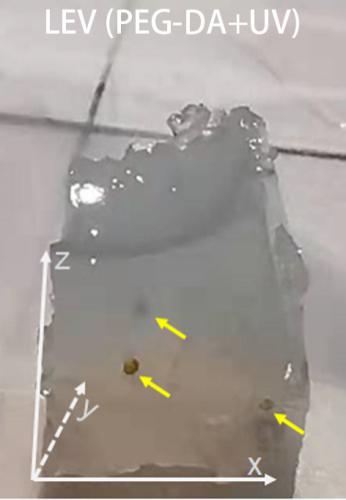


**Supplementary Figure 3.** Photo-polymerization of PEG-DA to freeze Arabidopsis seeds in magnetic levitation condition. Yellow arrows indicated levitated seeds in three-dimensional space (3D).


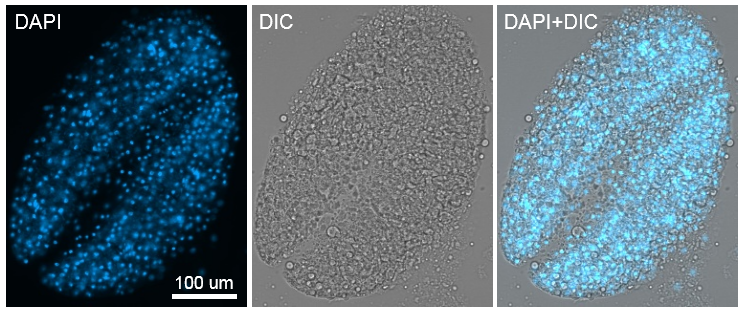


**Supplementary Figure 4.** Sections (20 μm thick) of frozen Arabidopsis seeds. The blue color is fluorescence from the DAPI dye and the bright field image is obtained from DIC on the optical microscope.

**Section 3. Optimization of auxin related treatment conditions**

To study auxin response of seed levitation, auxin donor (indole-3-acetic acid, IAA), auxin biosynthesis inhibitor (L-Kynurenine, L-Kyn) and auxin transportation inhibitor (1-naphthylphthalamic acid, NPA) were chosen. Application of these chemicals to the 3^rd^ primary root, 100 µg/L IAA was sufficient to statistically induce fluorescent signal of transgenic seedling DR5::GFP in root tip (Supplementary Figure 5A). And auxin responses further increased when more IAA was applied. While auxin response was unaffected by less dosage of NPA or L-Kyn, until the concentration reached 500 µg/L, respectively (Supplementary Figure 5B). The working concentrations of these auxin-related chemicals are optimized to initiate corresponding phenomenon, including IAA at 100 µg/L, NPA at 500 µg/L and L-Kyn at 500 µg/L respectively. Before artificial alteration of endogenous auxin level to study the effect of Gd^3+^-triggered microgravity on auxin response, the impact of Gd^3+^ on chemical-regulated auxin responses was analyzed. Compared to IAA-treated seeds, L-Kyn and NPA significantly prohibited fluorescence in DR5::GFP embryo sections (Supplementary Figure 6A). Statistically, Gd^3+^ showed limited impact on each chemical-treated condition (Supplementary Figure 6B). Moreover, the application of Gd^3+^ would not affect seed orientation in liquid without magnets (Supplementary Figure 6C). Together, auxin-related chemicals at indicated concentrations are appropriate to alter auxin level and compatible with Gd^3+^ for the study with the simulated microgravity condition.


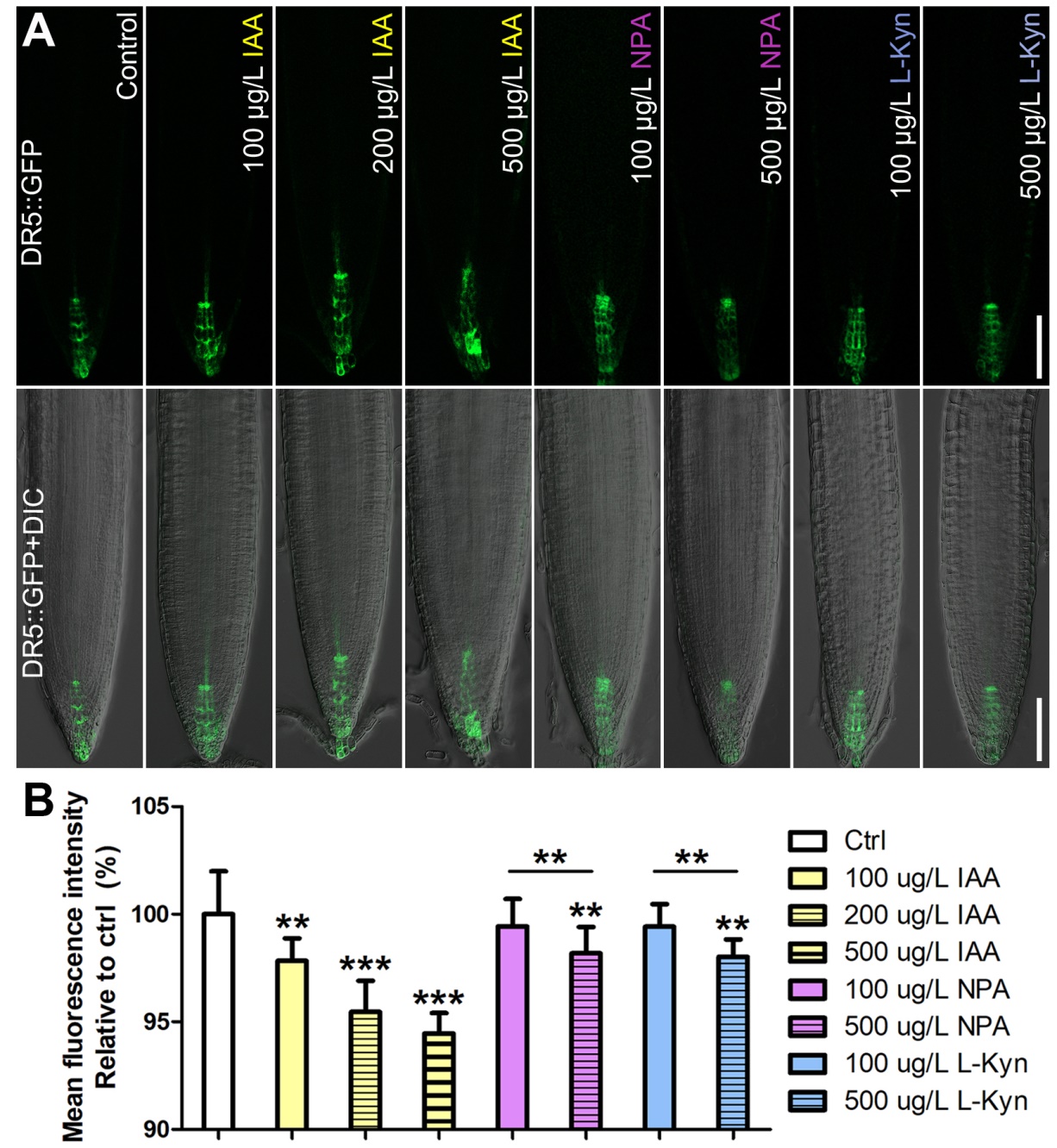


**Supplementary Figure 5.** (A) Root tips of DR5::GFP seedlings without (Ctrl) and with IAA, NPA or L-Kyn drug treatments at the indicated concentrations for 4 hours. The green color is the fluorescence of GFP. Scale bar = 100 μm. (B) Quantification on the expression of DR5::GFP in Arabidopsis root tips. Error bars represent SE of the mean. (n = 30, three biological repeats). Asterisks indicate significant differences (Student’s t-test, **, P ≤ 0.01, ***,P ≤ 0.001).


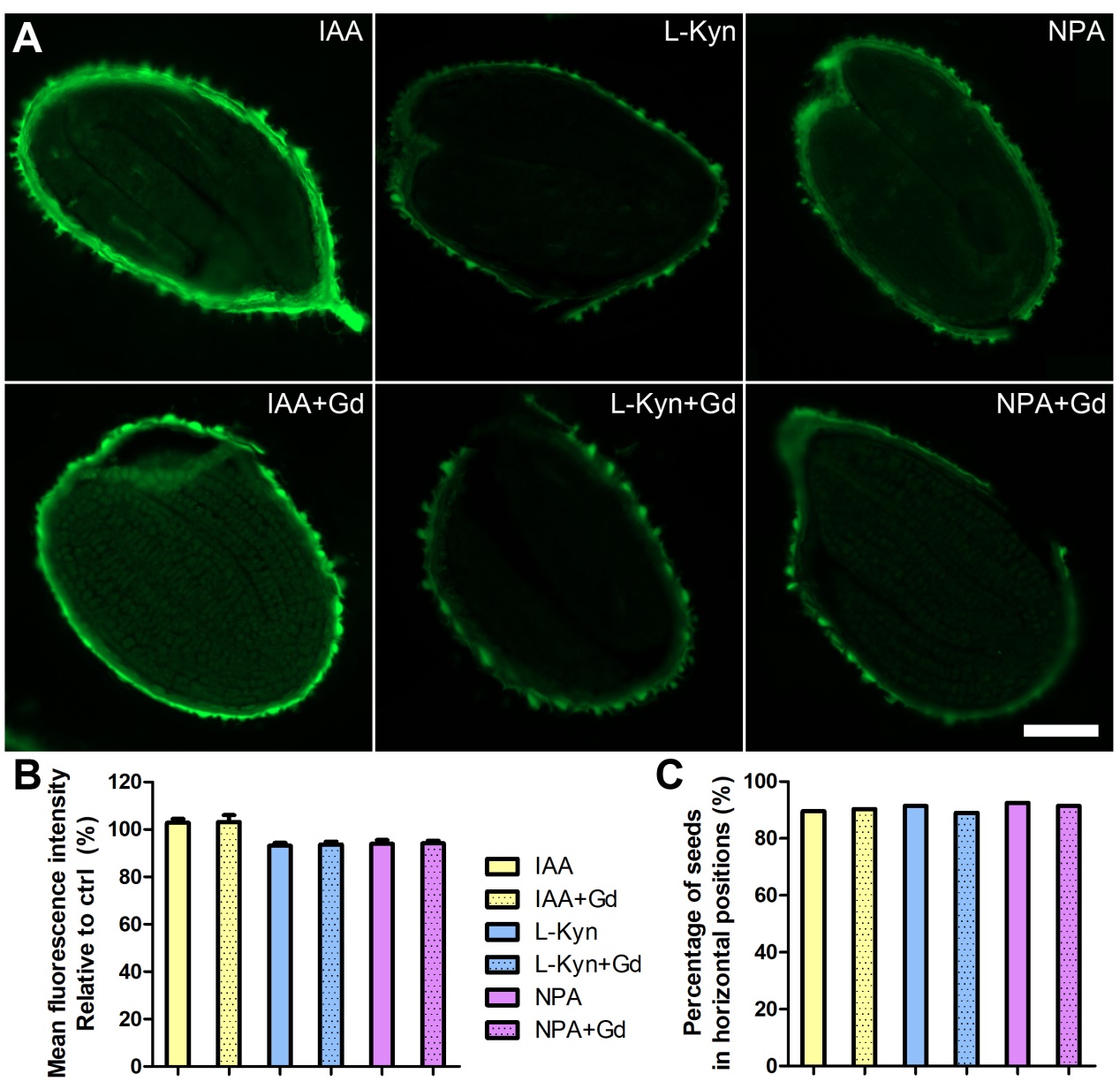


**Supplementary Figure 6.** Gd^3+^ shows limited impact on auxin response in auxin alteration conditions. (A) Expression of DR5::GFP in Arabidopsis embryo pretreated with auxin related drugs, following treatment without (IAA, L-Kyn, NPA) or with 200 mM Gd^3+^ solutions (IAA+Gd, L-Kyn+Gd, NPA+Gd). Scale bar = 100 μm. (B) Quantification on the expression of DR5::GFP in Arabidopsis embryo samples as defined in (A). Error bars represent SE of the mean. (n = 30, three biological repeats). (C) Quantification on the percentage of Arabidopsis seeds in 2D horizontal positions (n = 30).

**Section 4. Seed germination and seedling development**

After the short-term LEV treatment, the seeds were either sliced for auxin response study (as discussed in the paper), or transferred to normal gravity condition for the coming germination and seedling development. For those seeds transferred to normal gravity condition, the seeds were put in 1/2 MS medium in square petri dishes and kept for 6 days. The seedlings in petri dishes were scanned every day, and the root length of the samples was measured using ImageJ software and analyzed. The LEV-treated samples represented similar developmental phenotype as that in the control sample (Supplementary Figure 7A and 7C). While the germination is slightly faster in the LEV-treated sample within 24 hours, nevertheless, the difference disappeared in the following 24 hours (Supplementary Figure 7B), suggesting that LEV treatment represents impacts on auxin-related germination process but limited effects on the seedling development process.

Moreover, in order to study the effect of the long-term simulation of microgravity on seed germination and seedling development, a new microfluidic device with cultivation reservoirs specifically designed for long-period LEV treatment and seedling culturing should be developed and used to observe the whole process on-chip with various conditions of LEV-treatment in the near future.

**
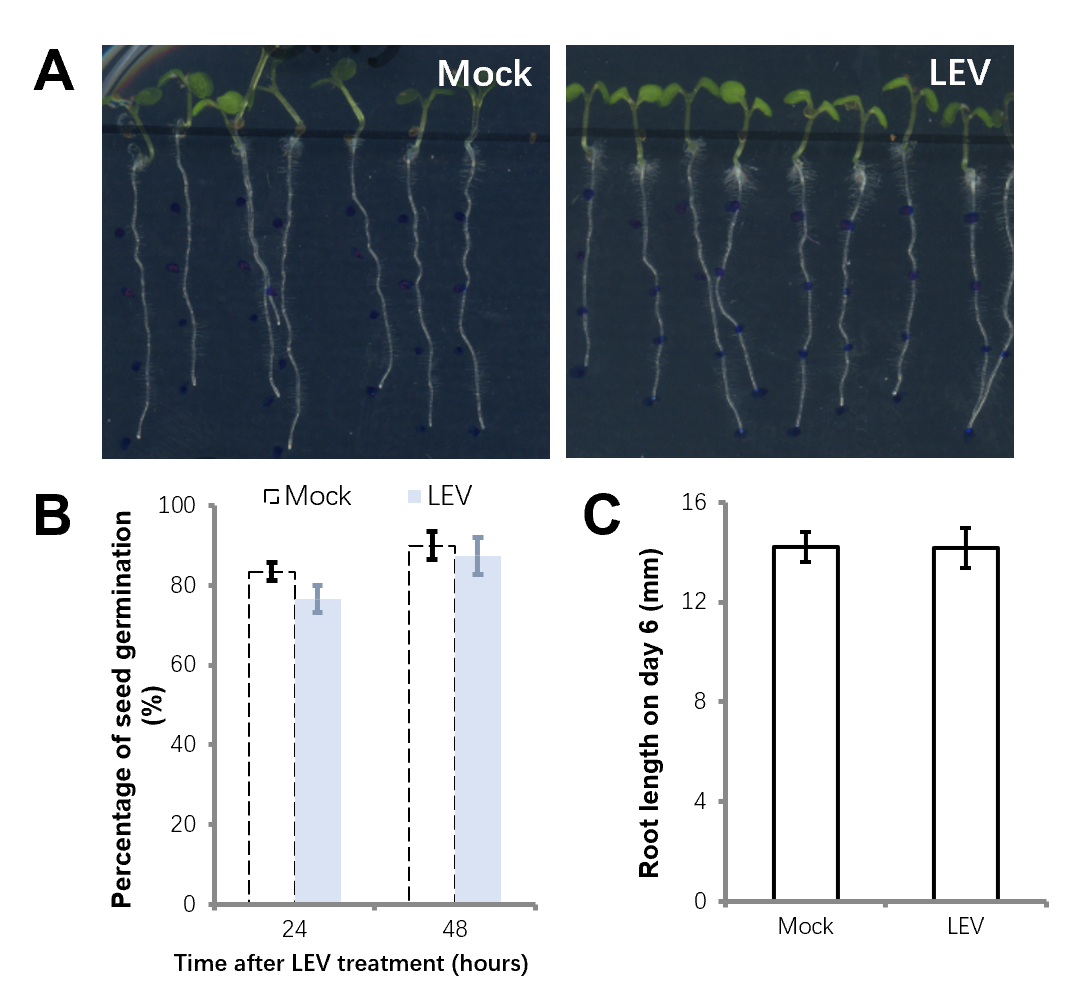
**

**Supplementary Figure 7.** Seedling growth and development after the LEV treatment. (A) 6-day-old seedlings with and without LEV treatment. (B) Percentage of seed germination at 24 and 48 hours. (C) Root length of seedlings on the 6th day after germination.

Supplementary Table 1. The density of Arabidopsis seeds

|  | 1000 seed weight  (g) | 1000 seed volume  (cm^3^) | Seed density  (g/cm^3^) |
| --- | --- | --- | --- |
| Group 1 | 123.5 | 101.1 | 1.2216 |
| Group 2 | 130.3 | 106.5 | 1.2235 |
| Group 3 | 134.9 | 110.5 | 1.2208 |
| Mean values±SE | 129.5667±4.6828 | 106.0333±3.8517 | 1.221967±0.001132 |

**References:**

1. Y. Luo, J. Liang, G. Zeng, M. Chen, D. Mo, G. Li, D. Zhang, Seed germination test for toxicity evaluation of compost: Its roles, problems and prospects. *Waste Management* **2018**, *71*, p109-114.

2. O. Biondi, S. Motta, P. Mosesso, Low molecular weight polyethylene glycol induces chromosome aberrations in Chinese hamster cells cultured in vitro. *Mutagenesis* **2002**, *17*, p261-264.
